# Takedown and Reuse of a Posterior Septal Nasal Floor Mucosal Flap for Skull Base Reconstruction in a Revision Surgery for a Recurrent Pituitary Tumor

**DOI:** 10.7759/cureus.35932

**Published:** 2023-03-09

**Authors:** Tomoharu Suzuki, Daisuke Murakami, Yusuke Miyamoto, Noritaka Komune, Takashi Nakagawa

**Affiliations:** 1 Otolaryngology - Head and Neck Surgery, Kyushu University, Fukuoka, JPN

**Keywords:** nasal mucosal flap, skull base surgery, cerebrospinal fluid leakage, endoscopic endonasal skull base surgery, posterior septal nasal mucosal flap

## Abstract

Various types of mucosal flaps can be used for skull base reconstruction after endoscopic endonasal skull base surgery (EESS). Preventing postoperative cerebrospinal fluid (CSF) leakage is essential. Flap creation during revision surgery can be problematic. We present a patient in whom a posterior septal nasal floor flap (PS-NF) was successfully reused for reconstruction after multiple reoperations for pituitary tumor resection.

A 22-year-old female underwent EESS for resection of a pituitary tumor and experienced multiple recurrences after repeated operations. For the third recurrence, a skull base surgery team comprising otolaryngologists and neurosurgeons performed a binostril combined transnasal/transseptal approach and used a PS-NF for reconstruction. For the fourth recurrence, a PS-NF was successfully taken down and reused for reconstruction. No postoperative CSF leakage or intranasal complications occurred.

Skull base reconstruction using a PS-NF is feasible and preserves the mucous membrane of the nasal septum and the morphology of the nasal cavity. PS-NF takedown and reuse is an option for revision EESS for recurrent pituitary tumors.

## Introduction

Endoscopic endonasal skull base surgery (EESS) for skull base lesions can be divided into three main phases: approach (securing surgical corridors), tumor removal, and skull base reconstruction. In our institution, otolaryngologists are mainly responsible for the approach and skull base reconstruction phases, while neurosurgeons perform the tumor removal.

Preventing postoperative cerebrospinal fluid (CSF) leakage is the primary goal of skull base reconstruction. Various pedicled mucosal flaps can be used and are selected according to the characteristics of each case. Use of a posterior septal nasal floor flap (PS-NF) preserves the anterior nasal septum mucosa and nasal structure better than the nasal septum flap (NSF) [1]. When revision surgery is being performed, the flap donor site and technique of flap creation for skull base reconstruction may be problematic. Although takedown and reuse of an NSF for reconstruction has been previously reported [2], to the best of our knowledge, takedown and reuse of a PS-NF has not. This report demonstrates that a PS-NF can be taken down and reused.

## Case presentation

A 22-year-old female with vision loss and bilateral hemianopsia owing to a pituitary adenoma producing adrenocorticotropic and growth hormones underwent endoscopic endonasal transsphenoidal resection by our neurosurgery department. One year later, reoperation for recurrence and loss of vision on the left was performed. Another reoperation was performed two years later, followed by intensity-modulated radiotherapy (50.4 Gy/28 fractions). Skull base reconstruction in the first three operations was performed by the neurosurgeons without using a pedicled mucosal flap.

**Figure 1:**
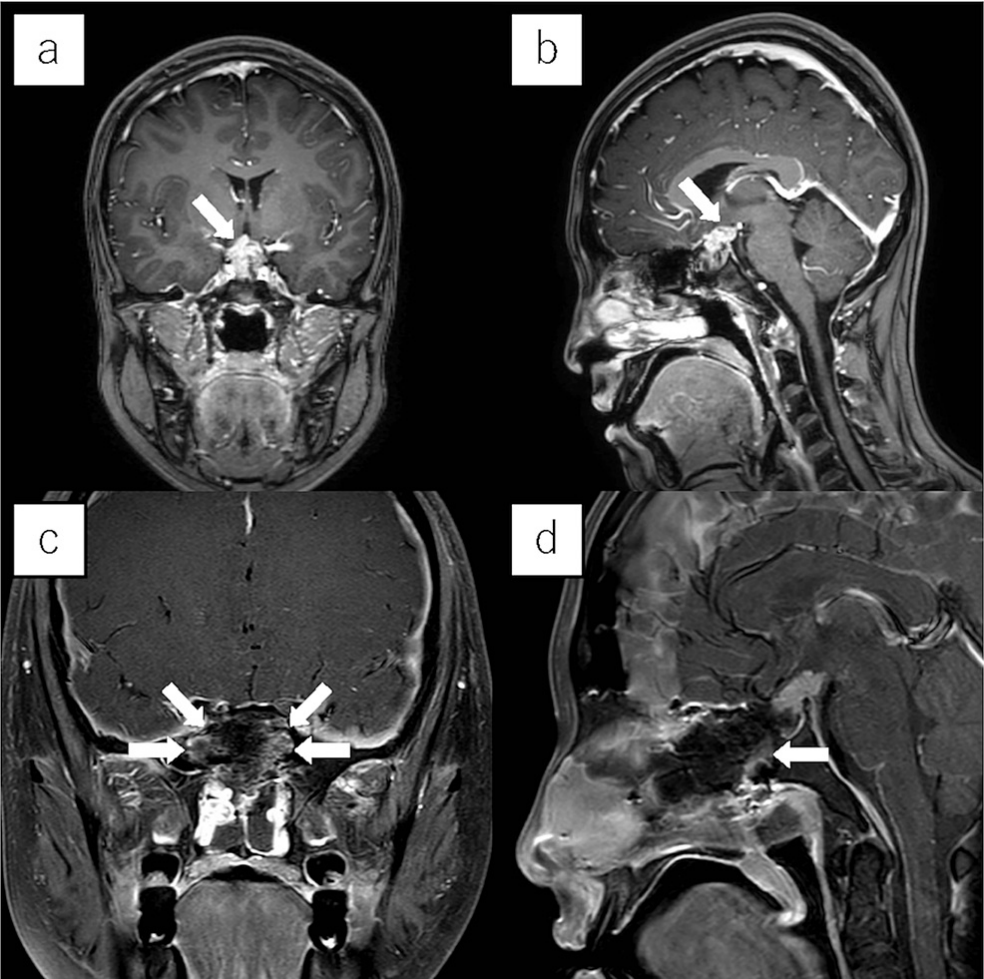
Pre- and postoperative contrast-enhanced MRI findings (a) Coronal and (b) sagittal contrast-enhanced magnetic resonance images before the fourth operation showed an enhancing 19 × 17 × 16 mm lesion within the sella that extended superiorly, dorsally, and to the right. The optic chiasm was mildly compressed anteriorly and superiorly. Tumor extension into the left cavernous sinus was suspected. The third ventricle was mildly compressed (white arrow). (c) Coronal and (d) sagittal images on the day after surgery showed contrast enhancement in the posterior septal nasal floor flap (white arrow); however, enhancement in the central region was minimal.

Five years after the third operation, another recurrence was found. On examination, corrected visual acuity was 20/200 on the right and 20/20 on the left. Quadrantanopia was noted on visual field testing. No other neurological deficits were apparent. Contrast-enhanced magnetic resonance imaging (MRI) showed a mildly enhancing 19 × 17 × 16 mm intrasellar lesion extending outside of the sella superiorly, dorsally, and to the right (Figure 1). The optic chiasm was mildly compressed anteriorly and superiorly. The third ventricle was also mildly compressed and tumor appeared to extend into the left cavernous sinus (Figures 1a-1b).

**Figure 2:**
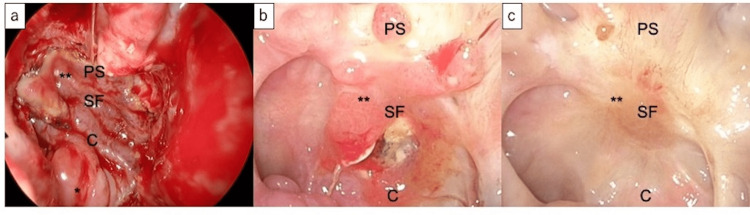
Intraoperative and postoperative fiberscopy findings (a) Endoscopic view of the opened sphenoid sinus after skull base reconstruction with a posterior septal nasal flap showed flap coverage of the sella floor and planum sphenoidale. (b) On fiberscopic examination 2 months after surgery, the crusts had almost disappeared and epithelialization in the central area was delayed. (c) Fiberscopic examination 5 months after surgery showed complete epithelialization. C, clivus; SF, sella floor; PS, planum sphenoidale; *, vascular pedicle; **, posterior septal nasal floor flap.

EESS was performed under general anesthesia by our skull base surgery team using a binostril combined transnasal/transseptal approach to preserve the nasal septum mucosa on one side [1]. The sellar floor was opened wide laterally until the internal carotid artery was exposed superiorly to the tuberculum sellae. The tumor was then removed en bloc in conjunction with the pituitary by the neurosurgeons. The base of the third ventricle was opened during removal and high-flow CSF leakage was observed. The skull base was reconstructed by inlaying the fascia lata and suturing it to the dura, rigidly reconstructing the ethmoid bone using a vertical plate, and using a right side of PS-NF for coverage (Figure 2a). The reconstructed area was then sealed with fibrin glue. Finally, the sphenoid sinus and nasal cavity were packed. The packing was removed on postoperative day seven. A lumbar drain was not placed. Figure 2 shows intraoperative and postoperative fiberscopy images.

Contrast-enhanced MRI on the day after surgery showed enhancement within the PS-NF but the enhancement in the central region was minimal (Figures 1c-1d). No complications, including postoperative CSF leakage, were observed. Fiberscopic examination two months after surgery showed that the crusts had almost disappeared but epithelialization in the central area was delayed (Figure 2b). Five months after surgery, the central area was completely epithelialized (Figure 2c). 

**Figure 3:**
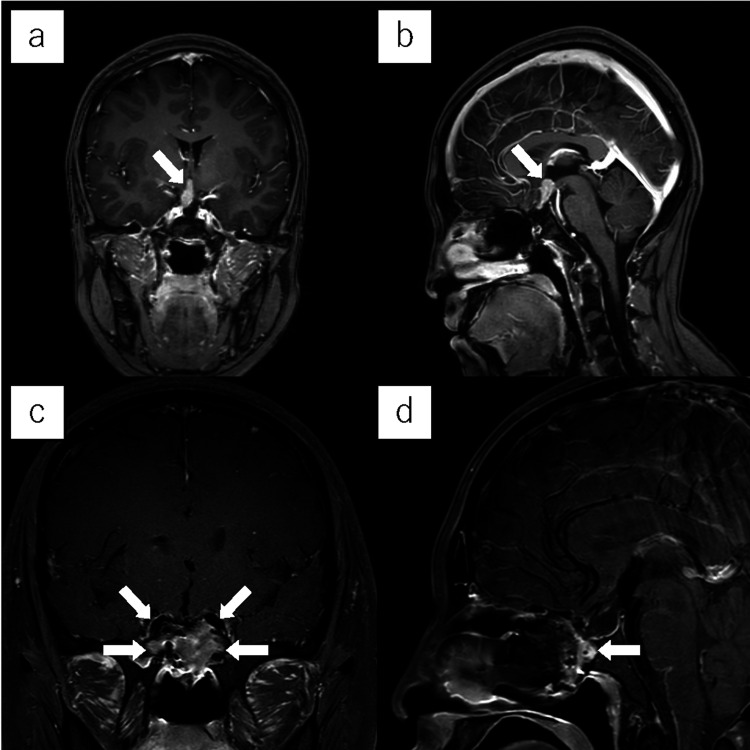
Pre- and postoperative contrast-enhanced MRI at the time of reoperation (a) Coronal and (b) sagittal contrast-enhanced magnetic resonance imaging before the fifth operation showed an enhancing 8 × 7 × 19 mm lesion to the right of the suprachiasmatic area extending to the third ventricle floor (white arrow). (c) Coronal and (d) sagittal images on the day after surgery showed complete tumor removal and sufficient enhancement of the posterior septal nasal floor flap (white arrows).

Postoperative contrast-enhanced MRI 18 months after surgery showed residual tumor within the third ventricle. Although she remained neurologically stable, serial imaging showed an increase in tumor size over time; therefore, the patient underwent a fifth surgery two years after the previous operation (Figure 3). Preoperative contrast-enhanced MRI showed an enhancing 8 × 7 × 19 mm lesion above the sella and posterior to the optic chiasm that extended to the floor of the third ventricle (Figures 3a-3b).

**Figure 4:**
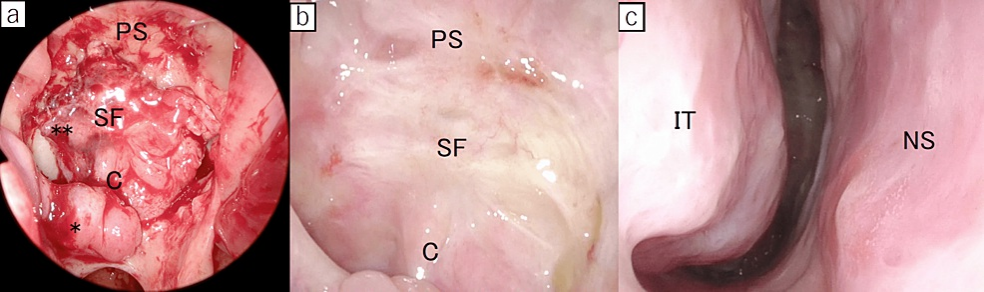
Intraoperative and postoperative fiberscopy findings at the time of reoperation (a) Endoscopic view of the opened sphenoid sinus after skull base reconstruction with a reused posterior septal nasal flap shows coverage of the sella with the flap. (b) Fiberscopic examination 3 months after surgery shows complete epithelialization. (c) Fiberscopic examination 3 months after surgery showed adequate preservation of the nasal septum and good epithelialization. C, clivus; IT, inferior turbinate; NS, nasal septum; SF, sella floor; PS, planum sphenoidale;*, vascular pedicle; **, posterior sepal nasal floor flap.

Another binostril approach was performed by the skull base surgery team. In the nasal cavity, the anterior wall of the sphenoid sinus was opened widely and the operative field for approaching the sella from both nasal cavities was secured. The nasal septum mucosa preserved in the previous operation could be held as it was. To reuse the PS-NF used in the previous operation, a mucosal incision was made anterior to the plane of the sphenoid sinus and the flap was dissected underneath the periosteum. Although there was mild adhesion to the bone, elevation was possible. Dissection proceeded to the base of the sphenoid sinus and the flap was preserved. During tumor removal by the neurosurgeons, the floor of the third ventricle was opened and high-flow CSF leakage was again observed. The sellar cavity was filled with an autologous fat graft taken from the right thigh. After suturing the dura, the remaining opening in the dura was covered with artificial dura and the previously used PS-NF. The site was then sealed with fibrin glue (Figure 4a). The sphenoid sinus and nasal cavity were packed. The packing was removed on postoperative day seven. A lumbar drain was not placed. Figure 4 shows intraoperative and postoperative fiberscopy images at the time of reoperation.

Contrast-enhanced MRI on the day after surgery showed complete tumor removal and sufficient enhancement within the re-used PS-NF (Figures 3c-3d). In fact, the degree of flap enhancement was greater compared with after the previous operation. The patient experienced no postoperative CSF leakage or other complications and was discharged home on postoperative day 11. Fiberscopic examination three months after surgery showed complete epithelialization, and the nasal septum was adequately preserved. (Figures 4b-4c). No recurrence has occurred in the first six months after surgery.

## Discussion

In our institution, a skull base surgery team comprising both otolaryngologists and neurosurgeons was formed in August 2018. When EESS is performed, the otolaryngologists are mainly responsible for the approach and skull base reconstruction, while the neurosurgeons perform tumor removal. In general, skull base reconstruction after tumor resection has not been standardized because it is based on the extent of the skull base defect and requires flexibility. Reconstruction techniques for revision surgery are controversial.

**Figure 5:**
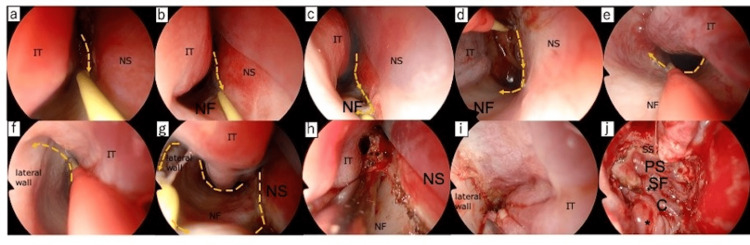
Procedure for creating PS-NF (a, b) An incision is made anteriorly from behind the nasal septum to the line of the anterior end of the inferior turbinate using a needle monopolar. (c) Then, the incision is continued to the floor of the nasal cavity. (d-f) Next, another incision is made anteriorly from the choana to the lateral wall of the inferior nasal meatus. (g) The incisions are connected at the lower front of the nasal septum. (h, i) The mucoperiosteal flap is elevated anterior-to-posterior using a suction elevator and then (j) along the arch of the choana to the sphenopalatine foramen while preserving the vascular pedicle. The flap is finally placed to fully cover the area from the sella to the planum sphenoidale. IT, inferior turbinate; NS, nasal septum; NF, nasal floor; SS, sphenoid sinus, C, clivus; SF, sella floor; PS, planum sphenoidale; *, vascular pedicle; **, posterior septal nasal floor flap

The PS-NF was first reported in 2014 as a pedicled mucosal flap substitute when an NSF cannot be used [3]. We have previously reported use of the PS-NF to reconstruct the sellar and parasellar regions as an alternative to the NSF [1]. The PS-NF can also be used for skull base reconstruction if high-flow CSF leakage is encountered during EESS [1,4]. The technique for PS-NF elevation is shown in Figure 5.

Various pedicled mucosal flaps can be used for skull base reconstruction based on the site of the skull base defect. The NSF is instrumental for covering large defects and when closure with a complementary nasal mucosal flap is difficult [5]. It is also useful when extensive skull base reconstruction is required if the contralateral nasal mucosa can be preserved. Although the NSF is associated with temporary crust formation, this does not cause long-term impairment of nasal septum function. However, if the mucosa does not epithelialize, there is a risk of cartilage necrosis and consequent nasal septum perforation; mucosal adhesions and olfactory disturbance have also been reported [6].

In our patient, a PS-NF was used instead of an NSF primarily because a PS-NF does not use the anterior nasal septal mucosa. Therefore, the risk of damaging the nasal septal cartilage and causing septum perforation is lower. We were able to preserve most of the nasal septum by protecting the nasal septum cartilage on the side of flap elevation and the nasal septum mucosa on the contralateral side. Even if a PS-NF becomes necrotic because of inadequate blood supply and CSF leakage occurs, the preserved nasal septal mucosa on the contralateral side can be used to elevate a PS-NF or NSF during revision surgery. Moreover, like an NSF, a PS-NF can sufficiently cover a sellar defect by itself and be reused for revision surgery, as shown in this case.

Although the width and length of a PS-NF are similar to those of an NSF, the total area of a PS-NF is slightly smaller; therefore, use of a PS-NF should be limited to coverage of skull base defects <3 cm in diameter if high-flow CSF leakage is encountered during surgery [7]. In our patient, high-flow CSF leakage was encountered and the skull base defect was <3 cm in diameter. A PS-NF was able to cover the sella to the planum sphenoidale adequately. Furthermore, our patient did not experience postoperative CSF leakage or intranasal complications and postoperative fiberscopic examination months after surgery showed complete epithelialization and preservation of nasal cavity morphology. Use of a PS-NF appears to have been appropriate. 

To the best of our knowledge, this is the first report of takedown and reuse of a PS-NF for revision EESS. Reuse involves making a mucosal incision with a scalpel in front of the planum sphenoidale and elevating the mucoperiosteum using a suction elevator. Although mild adhesion to the bone was observed, elevation was relatively easy; however, a needle monopolar was used as needed. Use of a CO2 laser for reuse of an NSF has also been previously reported [8]. The method of elevation should be considered on an individual case-by-case basis.

One caution when reusing a flap is that the initial flap size may be smaller and not adequately cover the skull defect; a larger flap that adequately covers the sella and sphenoid sinus should be created. In our patient, the initial flap (fourth operation) was large enough to cover the planum sphenoidale; however, in the fifth operation, only the sella floor required coverage. The reused flap would not have reached the planum sphenoidale because of size reduction. In addition, other reconstructive materials should be used if the flap is too small or flap blood flow is deemed insufficient. Flap hemodynamics can be evaluated using intraoperative indocyanine green angiography [7], which we are considering using in the future. Interestingly, contrast enhancement of the flap was more pronounced in the second flap. We hypothesize that the delay phenomenon (or “ischemic preconditioning”) caused by flap re-elevation was responsible [9].

## Conclusions

Skull base reconstruction using a PS-NF is feasible when performing EESS and preserves the mucous membrane of the nasal septum and the morphology of the nasal cavity. PS-NF takedown and reuse is an option for revision surgery for recurrent pituitary tumors.
